# Ablation of GSDMD Attenuates Neurological Deficits and Neuropathological Alterations After Traumatic Brain Injury

**DOI:** 10.3389/fncel.2022.915969

**Published:** 2022-05-20

**Authors:** Hao Du, Chang-Hong Li, Ruo-Bing Gao, Xiao-Qing Cen, Ping Li

**Affiliations:** ^1^Department of Army Occupational Disease, State Key Laboratory of Trauma, Burns and Combined Injury, Research Institute of Surgery and Daping Hospital, Army Medical University (Third Military Medical University), Chongqing, China; ^2^College of Bioengineering, Chongqing University, Chongqing, China; ^3^Institute of Brain and Intelligence, Army Medical University (Third Military Medical University), Chongqing, China

**Keywords:** GSDMD, NLRP3 inflammasome, traumatic brain injury, neuroinflammation, neuropathology

## Abstract

Pyroptosis plays a significant role in neuroinflammation after traumatic brain injury (TBI). However, the role of pyroptosis executor Gasdermin D (GSDMD) in neurological deficits and neuropathological alterations after TBI have not been elucidated. Our results demonstrated that GSDMD-KO exerted striking neuroprotective effects on motor dysfunction and neuropathological alterations (loss of synaptic proteins, microglia activation, astrogliosis, dendrite injury, and neuron death) at 3 days after TBI. GSDMD-KO inhibited the expression and release of pro-inflammatory cytokine releases (IL-1β and TNF-α) while promoting those of anti-inflammatory cytokines (IL-10 and TGF-β1). The temporal pattern of diverse inflammasome signals showed long-lasting elevations of NLRP3, caspase 1, and caspase 1 p20 after TBI, rather than NLRP1, NLRC4 or AIM2, similar to the change in GSDMD postinjury; and NLRP3-KO not only inhibited the expression and cleavage of GSDMD but also attenuated the loss of synaptic proteins and neurological deficits. Notably, RNA sequencing showed both GSDMD-KO and NLRP3-KO reversed the global expression of neuroinflammation- and neuropathology-related genes after TBI. Our findings proved that the inhibition of GSDMD exerts neuroprotective effects after TBI and is mainly driven by the NLRP3 inflammasome. GSDMD serves as a potent therapeutic target for the treatment of TBI.

## Introduction

Traumatic brain injury (TBI) is a great challenge that imposes heavy social and economic burdens worldwide and affects more than 50 million people each year (Jiang et al., 2019). TBI is considered a double-phase injury characterized by a primary injury caused by external forces and a delayed secondary injury (O Brien et al., 2020). The long-term outcomes of TBI are mainly determined by secondary injury, which has drawn extensive attention from researchers. It has been demonstrated that diverse mechanisms contribute to the progression of secondary injury after TBI, including reactive oxygen species, mitochondrial dysfunction, blood brain barrier dysfunction, and neuroinflammation, while long-lasting neuroinflammation is considered the main contributor to the development of secondary injury (Corps et al., 2015; Morganti-Kossmann et al., 2019). Neuroinflammation development is implicated in numerous processes, including inflammatory cell infiltration and the release of inflammatory cytokines. The overactivation of neuroinflammation is considered the main reason for many of the complications that occur after TBI, such as motor dysfunction and cognitive deficits (Corps et al., 2015; Wilson et al., 2017). Therefore, the regulation of neuroinflammation after TBI has drawn extensive attention for TBI treatment.

Inflammasome signals have been implicated in the modulation of neuroinflammation in many central nervous system (CNS) diseases including Alzheimer’s disease (AD), Parkinson’s disease (PD), depression, and TBI (Ismael et al., 2021; Li et al., 2021). However, most of these studies focused on the most well-known NLRP3 inflammasome or the inflammasome signaling effector caspase 1 (Irrera et al., 2017; Liu et al., 2018), while they rarely paid attention to the pyroptosis executor gasdermin D (GSDMD).

GSDMD is a member of gasdermin family and has membrane pore-forming activity (Shi et al., 2017). The activation of inflammatory caspases (caspase 1/11 in mice or caspase 4/5 in humans) leads to the cleavage of full-length GSDMD at the Asp276 or Asp275 site in mice and humans, respectively, which then breaks the link between the two domains and releases the N-terminal domain (N-GSDMD) and C-terminal domain (C-GSDMD). The cleavage of this link abolishes the autoinhibition of the C-GSDMD domain on the N-GSDMD domain, and the latter then oligomerizes and binds to the cell membrane to form pores (Pandeya et al., 2019). It was newly found that the cleavage of GSDMD by inflammatory caspases (mainly caspase 1) is essential for pyroptosis and IL-1β secretion in mouse macrophages (He et al., 2015; Shi et al., 2015). Although it has been proven that GSDMD plays a crucial role in the ischemic stroke and experimental autoimmune encephalomyelitis mouse models (Li et al., 2019; Wang et al., 2020), the role of GSDMD in neuroinflammation progression after TBI has not been investigated.

The cleavage of GSDMD is driven by diverse inflammasome signals, which share the same downstream effector caspase 1 in the canonical pathway (Shi et al., 2017; Burdette et al., 2021). Moreover, it has been reported that multiple inflammasomes, including NLRP1, AIM2, NLRP3, and NLRC4, participate in the progression of neuroinflammation after TBI and that directly or indirectly targeting inflammasome signaling could mitigate neuroinflammation and contribute to better outcomes (de Rivero Vaccari et al., 2009; Ge et al., 2018; Sun et al., 2020). Nevertheless, these studies have not shown a temporal alteration of diverse inflammasomes after TBI or determined the main contributor among them. Because GSDMD is activated by many inflammasome signals, inhibition of a single inflammasome pathway may not be sufficiently effective. Therefore, we aim to explore the temporal changes in different inflammasomes after TBI and determine the dominant inflammasome pathway involved in neuroinflammation progression after TBI, which will enhance our understanding of the mechanisms of GSDMD-mediated neuroinflammation.

In this study, we investigated the temporal pattern of GSDMD and its localization after TBI. As the levels of GSDMD increased rapidly and dramatically postinjury, we aimed to investigate the neurobehavioral and neuropathological alterations in the early stage after TBI. Our results showed that GSDMD knockout (GSDMD-KO) attenuated neurological deficits and neuropathological alterations mainly by regulating inflammatory cytokine release at 3 days after TBI. Further studies confirmed that the NLRP3 inflammasome was the main contributor to the cleavage of GSDMD rather than other inflammasomes; and NLRP3 knockout (NLRP3-KO) also exerted neuroprotective effects similar to those of GSDMD-KO. Moreover, transcriptome RNA sequencing (RNA-seq) showed that both GSDMD-KO and NLRP3-KO reversed the global expression of neuroinflammation- and neuropathology-related genes. These results demonstrated GSDMD is a potent therapeutic target for TBI that is mainly driven by the NLRP3 inflammasome.

## Materials and Methods

### Animals

GSDMD^−/−^ mice (C57BL/6J background) were generated using CRISPR/Cas9 technique by the Cyagen Model Organisms company. Male and female heterozygous GSDMD^+/–^ mice were mated to obtain homozygous and littermate WT control mice. The genotype was identified using PCR, and the primers are shown in the Supplementary Tables S1, S2. The knockout out efficiency were verified by WB. Mice were housed in the animal center of Daping Hospital, Army Medical University [Certificate SCXK (Yu) 2002-0002, Chongqing, China]. All animal experiments were approved by the Laboratory Animal Welfare and Ethics Committee of the Army Medical University (AMUWEC20191822) and complied with the NIH Guide for the Care and Use of Laboratory Animals.

### TBI Model

The controlled cortical impact method was used to produce moderate TBI models with methods described previously (Li et al., 2008). Briefly, mice were anesthetized with intraperitoneal injection of 250 μl 2.5% Tribromoethanol (Sigma, T48402) and then subjected to a 5-mm-diameter craniotomy in the left parietal cortex. The center was placed between bregma and the lambdoid suture. An aerodynamic impact device (PSI, USA) with a 3-mm-diameter metal tip was used to produce the controlled cortical impact (2 mm below the dura, 3.5 m/s impact speed). Sham animals underwent the same procedure as TBI mice except for the impact.

### Western Blot (WB)

WB was performed as previously reported (Du et al., 2022). Briefly, brain tissues were lysed using RIPA buffer containing protease inhibitor cocktail (Thermo Scientific). The protein concentration was measured using a BCA kit (Solarbio). Proteins were denatured and electrophoresed with 10% stain-free SDS-PAGE gels (Bio-Rad). Total protein was visualized with Bio-Rad stain-free technology using a ChemiDoc imaging system (Bio-Rad). Then, the proteins were transferred to PVDF membranes and blocked for 1 h using quick-block solutions (Beyotime). Membranes were incubated with specific primary antibodies overnight at 4°C with gentle shaking. The antibodies used in the article and the dilutions were as follows: GSDMD (Abcam, ab219800), 1:1,000; N-GSDMD (CST, 10137), 1:1,000; PSD95 (Abcam, ab192757), 1:1,000; SYN1 (Abcam, ab254349), 1:1,000; SNAP25 (Abcam, ab109105), 1:1,000; VAMP1 (Abcam, ab151712), 1:1,000; NLRP1 (Santa Cruz, sc-390133), 1:200; NLRP3 (Adipogen, AG-20B-0014), 1:1,000; NLRC4 (Abcam, ab201792), 1:1,000; AIM2 (CST, 63660), 1:1,000; caspase 1 (Adipogen, AG-20B-0042), 1:1,000; caspase 1 p20 (CST, 89332), 1:1,000. The next day, the membranes were washed five times with TBST and incubated with specific horseradish peroxidase-conjugated secondary antibodies at 37°C for 1 h. Finally, the membranes were washed again and imaged by a ChemiDoc image system (Bio-Rad). Data were analyzed using ImageLab software (Bio-Rad).

### Immunofluorescence

Immunofluorescence was carried out as previously reported (Zhang et al., 2020). Coronal brain sections (30 μm) were made using a cryotome (Leica). The brain slices were washed with PBS and permeabilized using 0.3% Triton X-100. After that, 10% goat serum was used for slice blocking. Primary antibody solutions were made according to the dilution rate provided by manufacturers and applied to the slice incubations at 4°C overnight with gentle shaking. The primary antibodies and dilutions used in the article were as follows: GSDMD (Abcam, ab219800), 1:100; Iba-1 (Sigma, SAB2702364), 1:100; GFAP (CST, 3670), 1:200; NeuN (Millipore, MAB377), 1:100; CD68 (Abcam, ab125212), 1:500; GFAP (Abcam, ab7260), 1:300; MAP2 (Abcam, ab32454), 1:500. The next day, the slices were rewarmed at 37°C for 1 h and washed three times with PBS. Corresponding secondary antibodies (Abcam) were diluted and used for staining following by nuclear labeling using DAPI (10 μg/ml, Sigma). Finally, the slices were washed and mounted using antifade mounting medium (Santa). Then, the slices were imaged with confocal microscopy (Leica) and analyzed using ImageJ software (NIH).

### Behavioral Tests

As we aimed to explore neurofunctions at the early stage of TBI, we mainly examined changes in motor functions rather than cognition and learning memories, as they are affected by imbalanced motor functions. GSDMD-KO and NLRP3-KO mice as well as their littermate control mice were used for behavioral tests. All mice were chosen randomly and behavioral tests were performed by lab technicians who were blinded to the genotype and treatment of mice.

#### Neurological Severity Score (NSS) Test

Mice were scored for neurological severity at 3 days after TBI using the method reported before (Zeng et al., 2018). Neuromuscular function, performance on an inclined board, mobility, vestibulomotor function, and complex neuromotor functions were evaluated. The range of scores was from 0 to 20, with 0 for normal and 20 for the severest injury.

#### Beam Walk Test

The fine motor coordination of TBI mice was assessed by a beam walk test as described previously (Henry et al., 2020). Briefly, mice were placed on a 1-cm-width wooden beam, and the number of foot faults of the right hindlimb was recorded over 50 steps. Mice were trained for 3 days before sham or TBI and tested at 3 days after TBI.

#### Accelerating Rotarod Test

Gross motor function and balance were assessed on the accelerating rotarod as reported previously (Henry et al., 2020). Mice were placed on the rod, which accelerated from 4 to 40 RPM within 300 s. The latency to fall from the rod (or cling to and rotate with the rod) was recorded. Each mouse was given three trials at 3 days after TBI, and the latency times were averaged. A 10 min rest period with access to food and water was allowed between each trial.

#### Open Field Test

Spontaneous locomotor activity was assessed using the open field test as reported (Fan et al., 2017). The total distance traveled, and the times spent in the center area and in the perimeter were recorded across a 5-min recording period.

### Quantitative PCR (qPCR)

qPCR was performed as reported before (Du et al., 2022). Briefly, total RNA was extracted with an RNA isolation kit (Promega). The RNA purity and concentration were detected by a NanoDrop One UV spectrophotometer. Reverse transcription was conducted using a GoScript Reverse Transcription kit (Promega) according to the manufacturer’s instructions. cDNA was amplified using GoTaq qPCR mix (Promega) and the corresponding primers (Supplementary Table S3). The qPCR was performed for 40 cycles at 95°C for 15 s and 60°C for 1 min after an initial 10 min incubation at 95°C.

### Enzyme-Linked Immunosorbent Assay (ELISA)

Mouse IL-1β, TNF-α, IL-10, and TGF-β1 ELISA kits (Novus, Valukine VAL601, VAL609, VAL605, and VAL611) were used for the detection of IL-1β, TNF-α, IL-10, and TGF-β1 in the cortex lysates according to manufacturer’s instructions.

### RNA-Seq Data Analysis

Samples from the peri-injury and sham cortex of GSDMD-KO, NLRP3-KO, and WT mice were obtained and flash frozen in liquid nitrogen. Then, the samples were processed for RNA sequencing analysis at Shanghai Majorbio Biopharm Technology Company. Total RNA was extracted from the tissue using TRIzol Reagent and genomic DNA was removed using Dnase I (TaKara). The RNA quality was determined and only high-quality RNA sample (OD260/280 = 1.8–2.2, OD260/230≥2.0, RIN≥6.5, 28S: 18S≥1.0, >1 μg) was used for sequencing library construction. The RNA-seq transcriptome library was constructed using Truseq^TM^ RNA Sample Prep Kit (Illumina, FC-122-1002) and paired-end RNA-seq sequencing library was sequenced with Illumina HiSeq xten/NovaSeq 6000 sequencer (2 × 150 bp read length). The raw paired end reads were trimmed and quality controlled by SeqPrep^1^ and Sickle^2^ with default parameters. Then clean reads were separately aligned to reference genome (GRCm39) with orientation mode using HISAT2 software (Kim et al., 2015). The mapped reads of each sample were assembled by StringTie (Pertea et al., 2015). PCA were performed using the online Majorbio Cloud Platform^3^. Neuroinflammation- and neuropathology-related gene lists were downloaded from Nanostring.com, as they are widely used in the transcriptome analysis of CNS diseases and are well elucidated (Ising et al., 2019; Tsai et al., 2021). The neuroinflammation- (685 genes) and neuropathology- (703 genes) related genes were extracted from the total expression matrix and analyzed with R language (version 4.1.2; Supplementary File 2). The heatmaps were visualized using the pheatmap package^4^. The raw data have been uploaded to the SRA database under the accession number PRJNA820566.

### Statistics

Quantitative data are presented as the mean ± SEM. All data passed the normality test (Kolmogorov–Smirnov test) and were analyzed using one-way ANOVA followed by Tukey’s multiple comparisons test for three or more groups *via* GraphPad Prism 8. The *p* value < 0.05 was considered statistically significant.

## Results

### GSDMD Increased Significantly in the Peri-injury Cortex After TBI and Was Mainly Localized in Microglia

**Figure 1:**
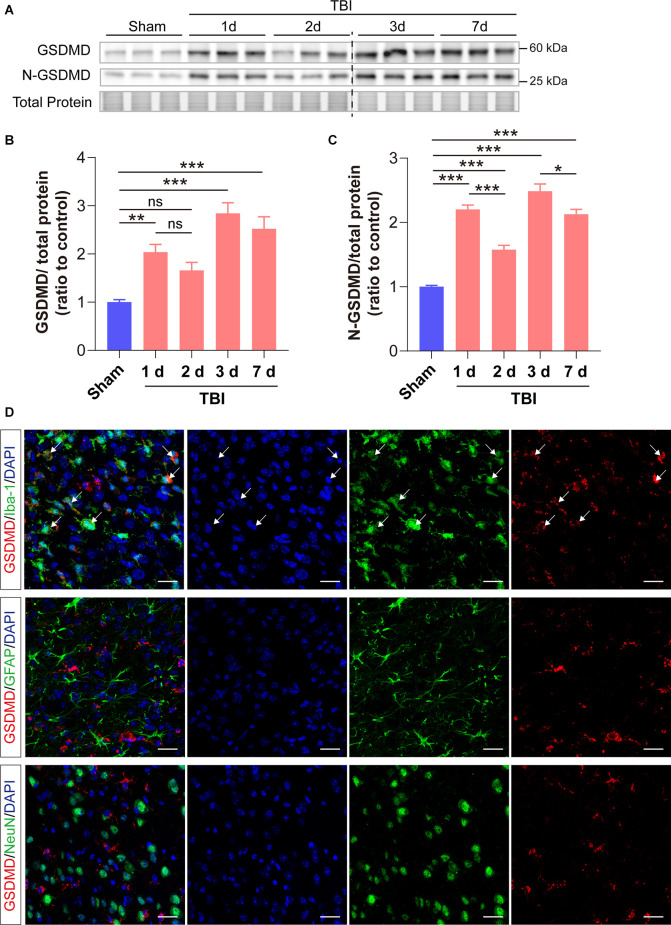
GSDMD increased significantly in the peri-injury cortex after TBI and was mainly localized in microglia. **(A)** Representative images of immunoblots for GSDMD, N-GSDMD, and total protein. **(B,C)** Quantitative analysis of GSDMD and N-GSDMD protein levels (*n* = 6). **(D)** Representative immunofluorescence showed the localization of GSDMD (green) with Iba-1, GFAP, and NeuN (red), in the peri-injury cortex at 3 days after TBI. Scale bar = 20 μm. White arrows indicate GSDMD^+^ and Iba-1^+^ cells. All data are presented as the mean ± SEM and were analyzed using one-way ANOVA followed by Tukey’s multiple comparisons test for significance. ns *p* > 0.05, *p < 0.05, **p < 0.01, and ***p < 0.001.

We first investigated the changes of GSDMD protein levels and its localization after TBI. Our results showed that the levels of GSDMD and N-GSDMD increased from 1 to 7 days after TBI and peaked at 3 days, while there was a small decrease at 2 days after TBI (Figures 1A–C). Moreover, immunofluorescence demonstrated that GSDMD was mainly localized in microglia (Figure 1D). These data suggested that microglial GSDMD participated in the neuroinflammation progression in the early stage of TBI; therefore, we aimed to explore the neurobehavioral and neuropathological changes during this stage postinjury.

### GSDMD-KO Attenuated Neurological Deficits and Neuropathological Alterations in the Peri-injury Cortex at 3 Days After TBI

**Figure 2:**
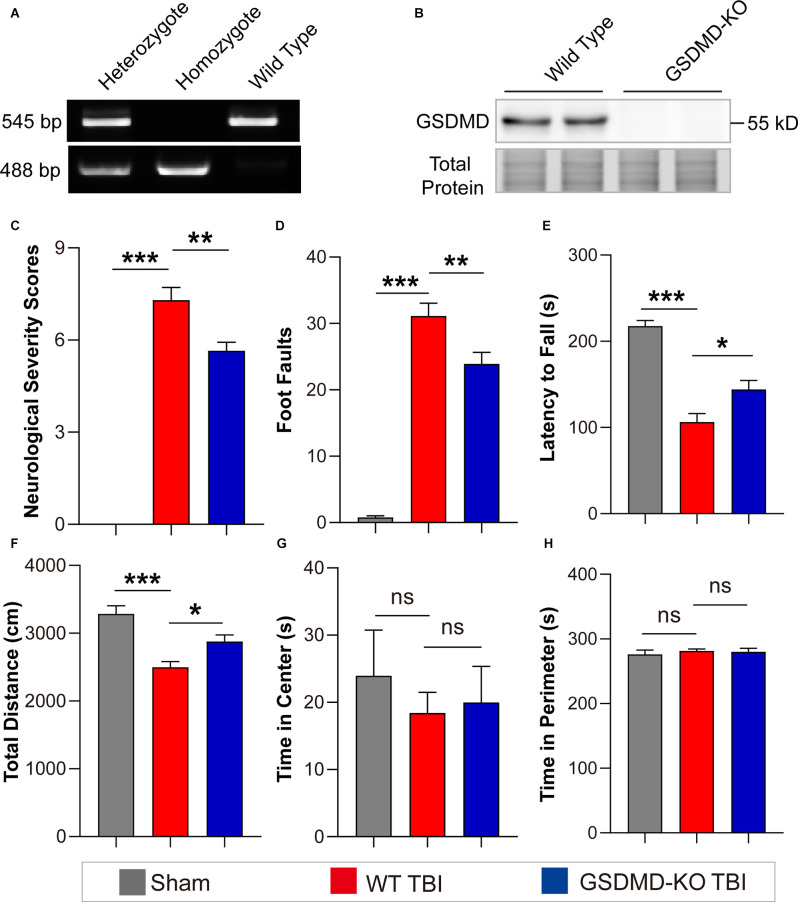
GSDMD-KO attenuated neurological deficits at 3 days after TBI. **(A)** Representative images of agarose gel electrophoresis for GSDMD-KO mouse genotyping. **(B)** Representative images of immunoblots for GSDMD using brain samples from WT mice and GSDMD-KO mice. **(C–E)** Quantitative analysis of NSS scores, foot faults, and latency to fall (s) from NSS test, beam walk test, and accelerating rotarod test at 3 days after TBI (*n* = 9–10). **(F–H)** Quantitative analysis of total distance (cm), time in center (s), and time in perimeter (s) in the open field test at 3 days after TBI (*n* = 9–10). All data are presented as the mean ± SEM and were analyzed using one-way ANOVA followed by Tukey’s multiple comparisons test for significance. ns *p* > 0.05, *p < 0.05, **p < 0.01, and ***p < 0.001.

As the levels of GSDMD and N-GSDMD peaked at 3 days after TBI, neurobehavioral tests were performed at this timepoint. GSDMD-KO was verified by PCR and WB (Figures 2A,B). The neurofunctions of GSDMD-KO and wild-type (WT) mice were examined with neurobehavioral tests used in this study to exclude differences caused by gene knockout. The results showed that NSS scores were 0 and foot faults were fewer than 3 for all mice, and there were also no significant differences in the accelerating rotarod test and the open field test (Supplementary Figures S1A–D). Then, neurobehavioral tests were performed on mice after TBI or sham treatment. The results proved that GSDMD-KO mice had lower NSS scores and foot faults than WT mice after TBI in the NSS test and the beam walk test (Figures 2C,D). GSDMD-KO mice exhibited a higher latency to fall and total distance, while no significant differences in the time spent in the center or perimeter during the accelerating rotarod test and the open field test (Figures 2E–H). These results confirmed that GSDMD-KO alleviated neurological deficits at 3 days after TBI.

**Figure 3:**
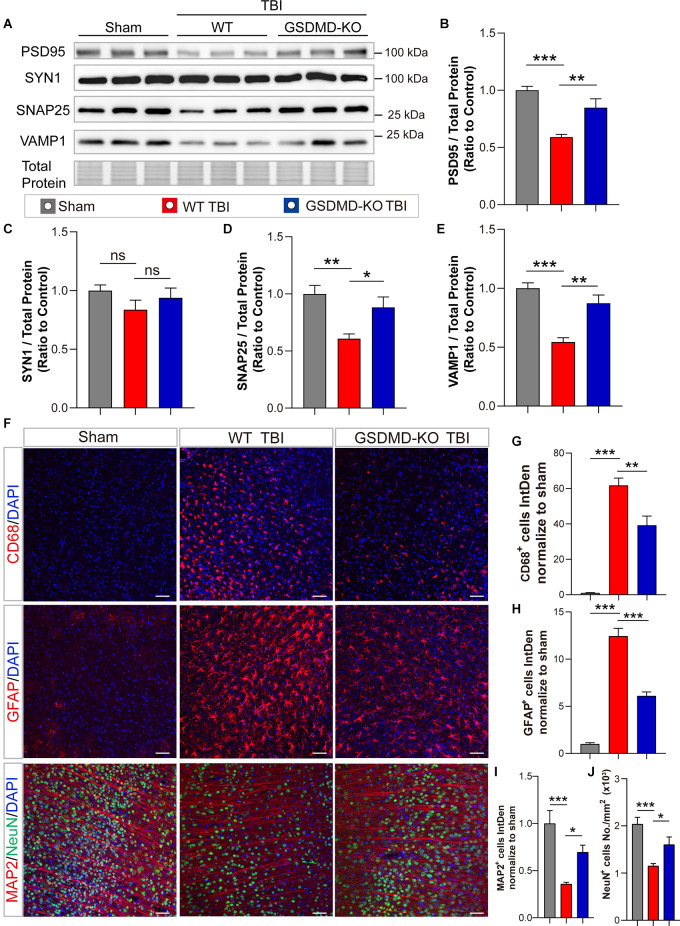
GSDMD-KO mitigated neuropathological alterations in the peri-injury cortex at 3 days after TBI. **(A)** Representative images of immunoblots for synaptic proteins (PSD95, SYN1, SNAP25, and VAMP1) and total protein. **(B–E)** Quantitative analysis of synaptic proteins (*n* = 6). **(F)** Representative immunofluorescence images of CD68, GFAP, MAP2, and NeuN in the peri-injury cortex after TBI or in the paired sham cortex. Scale bar = 50 μm. **(G–J)** Quantitative analysis of integrated intensity of CD68^+^, GFAP^+^, and MAP2 ^+^ cells and NeuN^+^ cell number/mm^2^ in the cortex (three slices from each brain and three brains were used for calculation). All data are presented as the mean ± SEM and were analyzed using one-way ANOVA followed by Tukey’s multiple comparisons test for significance. ns *p* > 0.05, *p < 0.05, **p < 0.01, and ***p < 0.001.

In addition, levels of synaptic proteins (PSD95, SNAP25, and VAMP1) from the peri-injury cortex were remarkably increased in GSDMD-KO mice compared to WT mice at 3 days after TBI, while there was no significant difference in the SYN1 protein level (Figures 3A–E). Moreover, as shown by immunofluorescence, GSDMD-KO reduced the levels of CD68^+^ cells and GFAP^+^ cells (Figures 3F–H) and enhanced the densities of MAP2^+^ cells and NeuN^+^ cells in the peri-injury cortex at 3 days postinjury (Figures 3F,I,J). These data demonstrated that GSDMD-KO could mitigate neuropathological alterations at 3 days after TBI.

### GSDMD-KO Diminished Pro-inflammatory Cytokine Release but Enhanced Anti-inflammatory Cytokine Release at 3 Days After TBI

**Figure 4:**
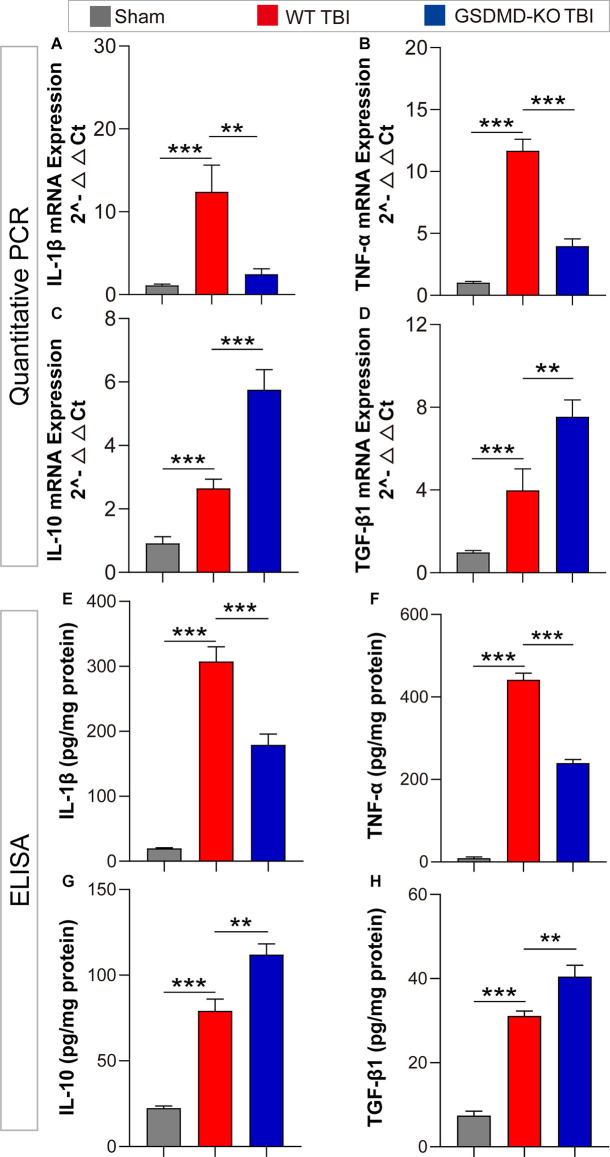
GSDMD-KO diminished pro-inflammatory cytokine release but enhanced anti-inflammatory cytokine release at 3 days after TBI. **(A–D)** Quantitative analysis of IL-1β, TNF-α, IL-10, and TGF-β1 mRNA levels detected by qPCR (*n* = 7–8). **(E–H)** Quantitative analysis of IL-1β, TNF-α, IL-10, and TGF-β1 levels in the cortex lysates detected by ELISA (*n* = 6). All data are presented as the mean ± SEM and were analyzed using one-way ANOVA followed by Tukey’s multiple comparisons test for significance. **p < 0.01 and ***p < 0.001.

As GSDMD plays an important role in the release of inflammatory cytokines, we investigated the effects of GSDMD-KO on the expression and release of pro-inflammatory (IL-1β and TNF-α) and anti-inflammatory (IL-10 and TGF-β1) cytokines. Our results showed that GSDMD-KO significantly reduced the expression and release of IL-1β and TNF-α while enhancing those of IL-10 and TGF-β1 in the peri-injury cortex at 3 days after TBI (Figures 4A–H). These findings confirmed that GSDMD exerts neuroprotective effects by regulating inflammatory cytokines.

### GSDMD Cleavage After TBI Was Mainly Driven by the NLRP3 Inflammasome Rather Than the NLRP1, NLRC4, and AIM2 Inflammasomes

**Figure 5:**
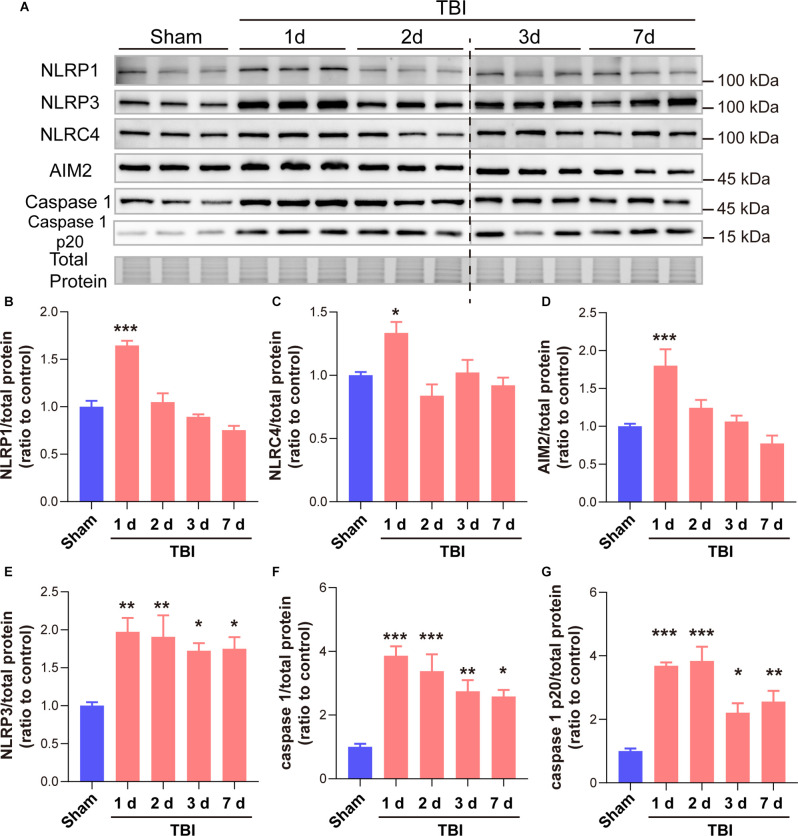
GSDMD cleavage after TBI was mainly driven by the NLRP3 inflammasome rather than the NLRP1, NLRC4, and AIM2 inflammasomes. **(A)** Representative immunoblots images of NLRP1, NLRP3, NLRC4, AIM2, caspase 1, caspase 1 p20, and total protein. **(B–G)** Quantitative analysis of NLRP1, NLRC4, AIM2, NLRP3 caspase 1, and caspase 1 p20 levels (*n* = 6). All data are presented as the mean ± SEM and were analyzed using one-way ANOVA followed by Tukey’s multiple comparisons test for significance. *p < 0.05, **p < 0.01, and ***p < 0.001.

As many types of inflammasomes participate in the activation and cleavage of GSDMD, we then tested the temporal patterns of diverse inflammasome proteins, including NLRP1, NLRP3, NLRC4, and AIM2, as well as the downstream co-effector caspase 1 and caspase 1 p20. Our results showed that NLRP1, NLRC4, and AIM2 only increased at 1 day after TBI while no significant differences at 2 days to 7 days after TBI (Figures 5A–D). However, long-term elevations in NLRP3, caspase 1, and caspase 1 p20 were found from 1 day to 7 days after TBI (Figures 5E–G). These data demonstrated that the temporal expression patterns of NLRP3, caspase 1, and caspase 1 p20 were similar to those of GSDMD and N-GSDMD after TBI, rather than NLRP1, AIM2, and NLRC4, suggesting that the NLRP3 inflammasome pathway mainly contributes to the activation of GSDMD after TBI.

### NLRP3-KO Inhibited the Expression and Cleavage of GSDMD and Attenuated Synaptic Protein Loss and Neurological Deficits at 3 Days After TBI

**Figure 6:**
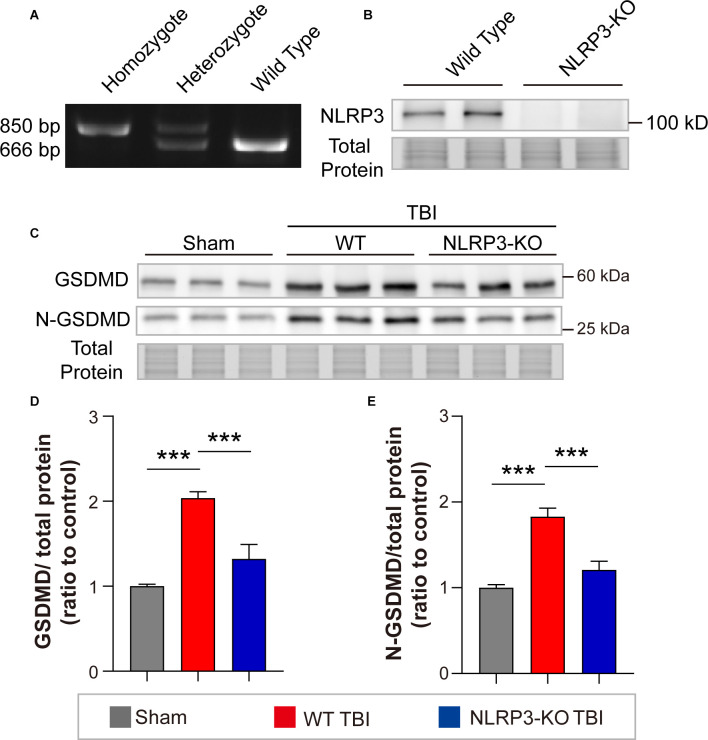
NLRP3-KO inhibited the expression and cleavage of GSDMD at 3 days after TBI. **(A)** Representative images of agarose gel electrophoresis for NLRP3-KO mouse genotyping. **(B)** Representative images of immunoblots for NLRP3 using brain samples from WT mice and NLRP3-KO mice. **(C)** Representative images of immunoblots for GSDMD, N-GSDMD, and total protein. **(D,E)** Quantitative analysis of GSDMD and N-GSDMD levels (*n* = 6). All data are presented as the mean ± SEM and were analyzed using one-way ANOVA followed by Tukey’s multiple comparisons test for significance. ***p < 0.001.

To interrogate whether NLRP3 regulates GSDMD after TBI and its contributions to outcomes postinjury, we further investigated the effects of NLRP3-KO on the expression and cleavage of GSDMD, loss of synaptic proteins (PSD95, SYN1, SNAP25, and VAMP1) and neurological functions after TBI. The NLRP3-KO efficiency was verified by PCR and WB (Figures 6A,B). Our results showed that NLRP3-KO reduced the levels of GSDMD and N-GSDMD at 3 days after TBI (Figures 6C–E).

**Figure 7:**
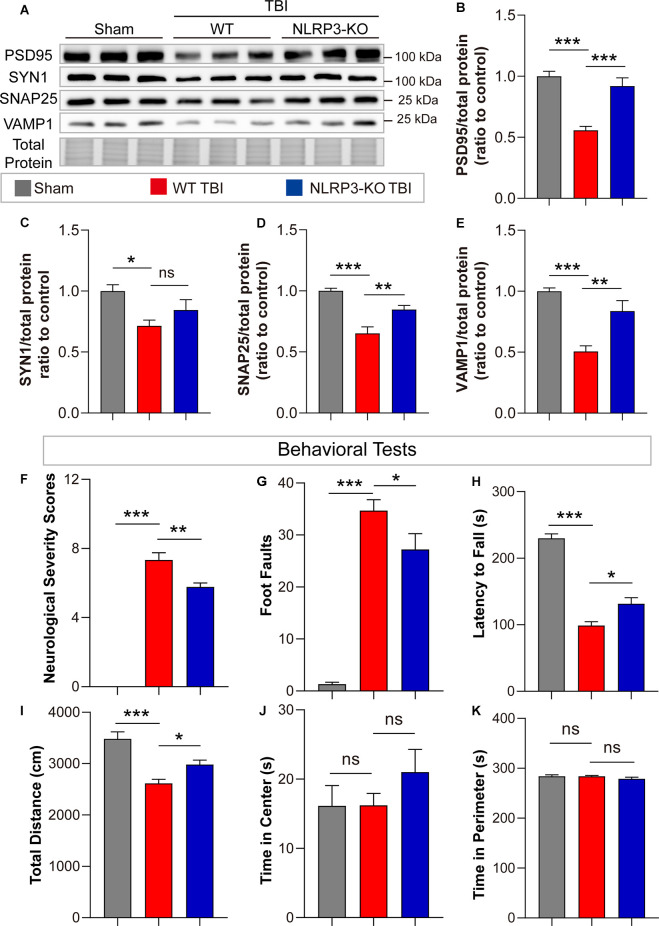
NLRP3-KO attenuated synaptic protein loss and neurological deficits at 3 days after TBI. **(A)** Representative images of immunoblots for synaptic proteins (PSD95, SYN1, SNAP25, and VAMP1) and total protein. **(B–E)** Quantitative analysis of synaptic proteins (*n* = 6). **(F–H)** Quantitative analysis of NSS scores, foot faults and latency to fall (s) from the NSS test, beam walk test, and accelerating rotarod test (*n* = 9–10). **(I–K)** Quantitative analysis of total distance (cm), time in center (s) and time in perimeter (s) in the open field test (*n* = 9–10). All data are presented as the mean ± SEM and were analyzed using one-way ANOVA followed by Tukey’s multiple comparisons test for significance. ns *p* > 0.05, *p < 0.05, **p < 0.01, and ***p < 0.001.

In addition, remarkable increases in synaptic proteins (PSD95, SNAP25, and VAMP1) were found in NLRP3-KO mice compared to WT mice after TBI, but there was no significant difference in SYN1 protein levels (Figures 7A–E). The neurofunctions of NLRP3-KO and wild-type (WT) mice were examined and the results showed no significant differences between them (Supplementary Figures S1E–H). Then neurobehavioral tests were performed on mice after TBI or sham treatment. The results demonstrated NLRP3-KO TBI group showed lower NSS scores and foot faults than the WT TBI group (Figures 7F,G). Increasing latency to fall and total distance were observed in NLRP3-KO mice compared to WT mice after TBI, while there were no differences in the time spent in center or perimeter (Figures 7H–K). These results showed that NLRP3-KO exerts neuroprotective effects similar to those of GSDMD-KO by mitigating the expression and cleavage of GSDMD, which further confirmed that GSDMD cleavage is mainly driven by the NLRP3 inflammasome after TBI.

### Both GSDMD-KO and NLRP3-KO Reversed the Global Expression of Neuroinflammation- and Neuropathology-Related Genes at 3 Days After TBI

**Figure 8:**
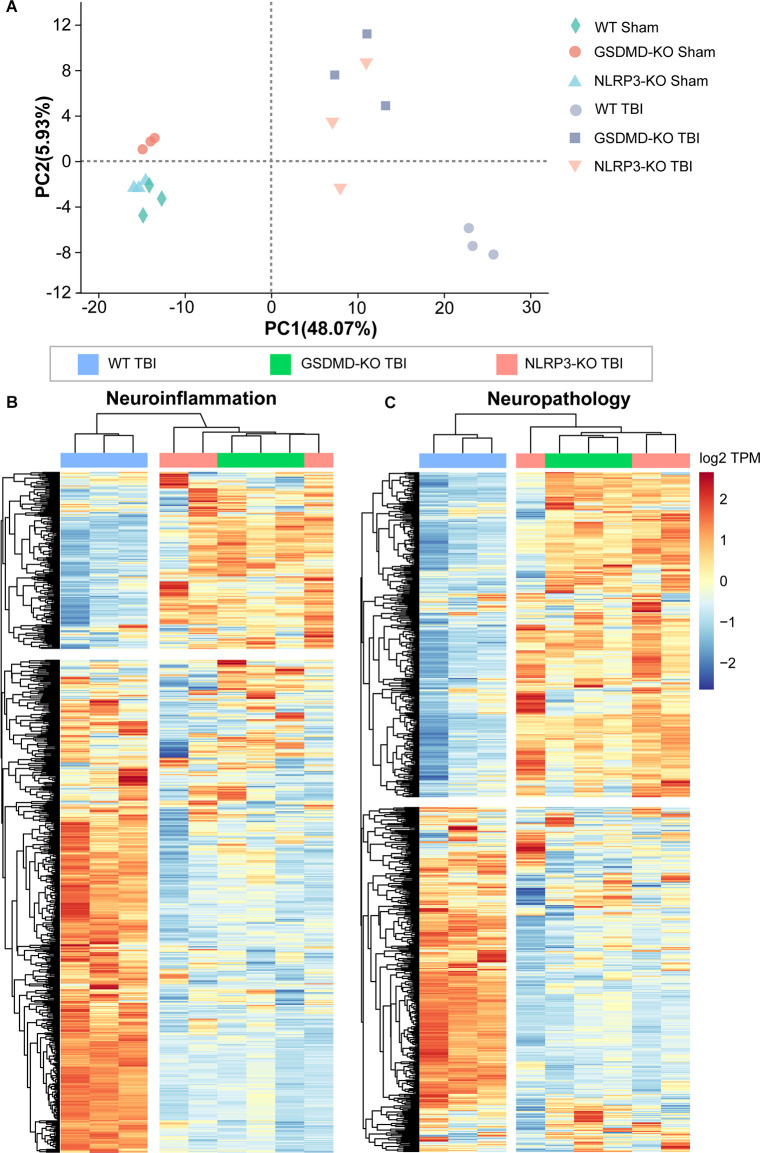
Both GSDMD-KO and NLRP3-KO reversed the global expression of neuroinflammation- and neuropathology-related genes at 3 days after TBI. **(A)** PCA analysis of RNA-seq data from WT, GSDMD-KO, and NLRP3-KO mice at 3 days after sham or TBI treatment (*n* = 3). **(B,C)** Heatmap analysis of neuroinflammation- and neuropathology-related gene expression patterns from WT, NLRP3-KO, and GSDMD-KO mice at 3 days after TBI.

We performed transcriptome RNA-seq to investigate the effects of GSDMD-KO and NLRP3-KO on the global expression of neuroinflammation- and neuropathology-related genes at 3 days after TBI. Principal component analysis (PCA) showed that the gene expression patterns of GSDMD-KO and NLRP3-KO mice were similar to those of WT mice before TBI but were strikingly different after TBI (Figure 8A). Moreover, heatmaps of neuroinflammation- and neuropathology-related gene expression showed similar expression patterns among GSDMD-KO, NLRP3-KO, and WT mice before TBI (Supplementary Figure S2) but remarkable different patterns were showed in GSDMD-KO and NLRP3-KO mice compared with WT mice (Figures 8B,C). In addition, expression patterns of neuroinflammation- and neuropathology-related genes in GSDMD-KO and NLRP3-KO groups were clustered together, which proved the similar roles of them at this timepoint after TBI (Figures 8B,C). Our results demonstrated GSDMD-KO and NLRP3-KO could globally reverse the expressions of neuroinflammation- and neuropathology-related genes at 3 days after TBI and the two groups are clustered together, which suggested both of them are in the same pathway.

## Discussion

In this study, we demonstrated the crucial role of GSDMD in early stage after TBI. The expression and cleavage of GSDMD increased at 1 day after TBI and lasted until 7 days. However, there was a small decrease at 2 days after TBI, which might be due to the subsidence of mechanical damage-induced acute inflammation; and it is also found that in the cerebrospinal fluid from infants and children with severe TBI, NLRP3 protein levels peaked within 24 h after TBI and then decreased at 25–48 h while increasing again from 48 h (Wallisch et al., 2017). This quadratic trend suggests that inflammasome signaling participates in both primary and secondary injury after TBI. Moreover, we found that levels of GSDMD and N-GSDMD peaked at 3 days after TBI, similar to the levels of NLRP3 inflammasome as reported previously (Xu et al., 2018). A previous study showed that many markers of M1 and M2 phenotype microglia/macrophage peaked at 3–5 days after TBI (Wang et al., 2013). Meanwhile, we also found that GSDMD mainly localized in microglia after TBI in the peri-injury cortex, which suggests that microglial GSDMD might be involved in microglial polarization. In general, our results illustrated the temporal pattern of GSDMD expression and cleavage as well as its localization, contributing to a better understanding of the crucial role of GSDMD in neuroinflammation progression after TBI.

GSDMD-KO showed striking neuroprotective effects on motor dysfunction at 3 days after TBI. It’s also reported that GSDMD-KO mice showed lower modified NSS scores than WT mice in an ischemic stroke animal model (Wang et al., 2020). A previous study showed that GSDMD-KO mice had lower clinical scores than their WT counterparts in an experimental autoimmune encephalomyelitis model (Li et al., 2019). These results proved that GSDMD plays an essential role in the neurofunctional impairment of CNS diseases and that its inhibition contributes to better outcomes. Moreover, abundant studies have reported that the inhibition of inflammasome signals protects against neurological deficits in TBI, PD, AD, subarachnoid hemorrhage, vascular dementia, etc. (Irrera et al., 2017; Ising et al., 2019; Poh et al., 2020; Rui et al., 2020; Hu et al., 2021). However, these studies usually reported one of the inflammasome signals. GSDMD is activated by diverse inflammasome signals and serves as the executor of pyroptosis, so the inhibition to GSDMD might result in better neurofunctional outcomes than single pathway inhibition.

The neuropathological alterations caused by TBI, including synaptic protein loss, microglial activation, astrogliosis, dendritic injury, and neuronal death were remarkably mitigated by GSDMD-KO. Some studies have reported that levels of synaptic proteins decreased after TBI at acute stage (1–7 days), which reflects the severity of injury (Feng et al., 2016; Rehman et al., 2018; Rosa et al., 2021); and pharmacological treatments that attenuated the loss of synaptic proteins protects against motor dysfunction post TBI (Rehman et al., 2018), which is also found in our study. Therefore, the change of synaptic proteins could reflect the motor dysfunction severity at acute stage post TBI. Moreover, the neuron death was mitigated by GSDMD-KO in our manuscript. There might be two reasons for this neuroprotective effect. First, GSDMD-KO attenuated neuroinflammation after TBI and therefore reduced the inflammation-induced neural apoptosis. Apoptosis also affects the development of neuroinflammation. So the inhibition of inflammasome signals also contribute to apoptosis attenuation. Second, NLRP3 is also expressed in neurons after TBI as reported before (Liu et al., 2013). Therefore, neuronal NLRP3 inflammasome activation might result in neuron pyroptosis directly. The attenuation of microglial activation, astrogliosis, and dendritic injury by GSDMD-KO also contribute to better neurofunction postinjury. Moreover, GSDMD-KO also regulated inflammatory cytokine release after TBI by diminishing pro-inflammatory cytokine release and promoting anti-inflammatory cytokine release. As it has been proven that the membrane pore-forming effects of GSDMD is essential for IL-1β secretion in macrophages (He et al., 2015), GSDMD also plays a pivotal role in the inflammatory cytokines release in CNS diseases, including TBI. The effects of GSDMD on inflammatory cytokine release regulation might be the main reason for reduced neuropathological changes after TBI in the GSDMD-KO mice.

We further investigated the temporal pattern of diverse inflammasome signals, the effects of NLRP3-KO on GSDMD activation and neurofunctional alterations. We finally confirmed that NLRP3 played a dominant role in the development of neuroinflammation after TBI rather than NLRP1, AIM2 or NLRC4 inflammasomes. It has been reported that NLRP1 knockout mice had no significant better outcomes than WT mice after TBI (Brickler et al., 2016). Some studies have shown that different inflammasomes increased after TBI and that targeting inflammasome signals *via* inhibiting the NLRP3 inflammasome pharmacologically or genetically, using caspase 1 inhibitors or anti-ASC antibodies, could attenuate neuroinflammation postinjury (de Rivero Vaccari et al., 2009; Irrera et al., 2017; Ge et al., 2018; Xu et al., 2018; Kuwar et al., 2019; Sun et al., 2020). However, a comprehensive understanding to the temporal pattern of diverse inflammasome signal activation after TBI is still needed to better understanding the role of inflammasome signals in neuroinflammation. We confirmed the expressions of NLRP3, caspase 1, and caspase 1 p20 elevated from 1 to 7 days after TBI and further proved the neuroprotective effects of NLRP3-KO postinjury. Moreover, RNA-seq analysis showed that expression patterns of neuroinflammation- and neuropathology-related genes in the GSDMD-KO TBI and the NLRP3-KO TBI groups were clustered together and both of them showed remarkable reversals to the global expression of neuroinflammation- and neuropathology-related genes. Our results demonstrated that the NLRP3-GSDMD pathway is the dominant inflammasome signal for neuroinflammation and neuropathology regulation after TBI.

In terms of the clinical translation of pyroptosis inhibition, more researches are still needed. First, the inhibition to NLRP3 protein directly usually aims to block the NACHT domain of NLRP3, including CY-09, Tranilast, Bay 11-7082, and MCC950 (Swanson et al., 2019; Li et al., 2021). This strategy is considered as the most efficient and specific way to control pyroptosis. However, as many of these chemical compounds are newly found so the clinical safety and side-effects in clinical translation should be considered. The clinical trial of the most well-known NLRP3 inhibitor MCC950 was suspended owing to hepatic toxicity (Swanson et al., 2019). In addition, other researchers aimed to develop caspase 1 inhibitors for the attenuation of inflammasome activation. Although the role of caspase 1 in cell death and inflammation have been demonstrated, emerging researches showed alternative caspase-independent pathway activated when performing caspase inhibition, which resulted in inadequate efficacy (Dhani et al., 2021); and the poor target specificity also limits the clinical application of caspase inhibitors (Kesavardhana et al., 2020). GSDMD was newly found as a pyroptosis executor and serves as a potent target for pyroptosis related diseases (Shi et al., 2015); and it is found that disulfiram, a drug used to treat alcohol addiction, could inhibit pyroptosis by blocking GSDMD pore formation (Hu et al., 2020). As this drug is almost approved by FDA and used in clinical practice, so the toxicity and side-effects are acceptable compared with other pyroptosis inhibitors. Other GSDMD inhibitors including necrosulfonamide and LDC7559 were also screened out, but there is a long way to develop them into clinical applications (Shi et al., 2017; Pandeya et al., 2019).

However, there are also limitations of our work. Although we aimed to investigate the effects of GSDMD on TBI-induced neurological deficits and neuropathological alterations in the early stage due to the high level of GSDMD at this time, the expression and cleavage of GSDMD is still higher at 7 days post TBI, so the long-term effects of GSDMD on TBI-induced brain damage including dementia and tauopathy should be investigated. It has been reported that the inflammasome activation and downstream IL-1β processing were found at 14 months post repetitive mild TBI and IL-1 receptor 1 knockout attenuated accumulation of pro-IL-1β and misfolded tau, thus protected against cognitive deficits post TBI (Wu et al., 2022). But the role of pyroptosis executor GSDMD on TBI-induced damage in the chronic stage remains elucidated. In our work, we only investigated the neuroprotective effects of GSDMD on TBI in the early stage, the long-term effects should be explored in the future.

## Conclusions

In this study, we investigated the temporal patterns of GSDMD expression and activation, which implicated that GSDMD participates in neuroinflammation progression in the early stage after TBI. Moreover, GSDMD-KO attenuated neurological deficits and neuropathological alterations after TBI and was mainly driven by the NLRP3 inflammasome pathway. Transcriptome RNA-seq showed that both GSDMD-KO and NLRP3-KO reversed the global expression patterns of neuroinflammation- and neuropathology-related genes at 3 days after TBI and their expression patterns were clustered together. Our studies shed new light on understanding the role of GSDMD in neuroinflammation regulation after TBI and provide a potent target for TBI therapy.

## Data Availability Statement

The datasets presented in this study can be found in online repositories. The names of the repository/repositories and accession number(s) can be found below: https://www.ncbi.nlm.nih.gov/, PRJNA820566.

## Ethics Statement

The animal study was reviewed and approved by the Laboratory Animal Welfare and Ethics Committee of the Army Medical University.

## Author Contributions

HD performed the construction of TBI mice model, western blots, immunofluorescence, quantitative analysis, RNA-seq data analysis, interpreted results, and wrote the manuscript. C-HL and R-BG performed mice genotyping, behavioral tests, and immunofluorescence. X-QC sampled mice, performed ELISA, and qPCR. PL conceived, designed, and supervised the study, interpreted results, and wrote the manuscript. All authors contributed to the article and approved the submitted version.

## Conflict of Interest

The authors declare that the research was conducted in the absence of any commercial or financial relationships that could be construed as a potential conflict of interest.

## Publisher’s Note

All claims expressed in this article are solely those of the authors and do not necessarily represent those of their affiliated organizations, or those of the publisher, the editors and the reviewers. Any product that may be evaluated in this article, or claim that may be made by its manufacturer, is not guaranteed or endorsed by the publisher.

## Footnotes

^1^ https://github.com/jstjohn/SeqPrep

^2^ https://github.com/najoshi/sickle

^3^ www.majorbio.com

^4^ https://cran.r-project.org/web/packages/pheatmap/index.html
