# Supplementary material for: Ablation of GSDMD Attenuates Neurological Deficits and Neuropathological Alterations After Traumatic Brain Injury
Source: Front Cell Neurosci. 2022 May 20;16:915969. doi: 10.3389/fncel.2022.915969 (PMC9164823; doi:10.3389/fncel.2022.915969)
Supplement: Supplementary file 2 [file Data_Sheet_1.PDF]

Supplementary table S1. Primers for GSDMD knockout mice genotype identification

number	primers	product size
F1	TCCACCGTTAGCCCAGAGAC	488 bp
R1	ACACCATAACAGTAGAAACCCTAAC	
F1	TCCACCGTTAGCCCAGAGAC	545 bp
R2	TTAGTTCCTCCAGCACTCAGG	

*Homozygotes: one band with 488 bp; Heterozygotes: two bands with 488 bp and 545 bp; Wildtype allele: one band with 545 bp

Supplementary table S2. Primers for NLRP3 knockout mice genotype identification

number	primers	product size
F1	TCAGTTTCCTTGGCTACCAGA	850 bp
R1	TTCCATTACAGTCACTCCAGATGT	666 bp
F2	TGCCTGCTCTTTACTGAAGG	

*Homozygotes: one band with 850 bp; Heterozygotes: two bands with 850 bp and 666 bp; Wildtype allele: one band with 666 bp

Supplementary table S3. Primers used for qPCR.

Gene	Primer
GAPDH	Forward: CTTTGTCAAGCTCATTTCCTGG
	Reverse: TCTTGCTCAGTGTCCTTGC
IL-1β	Forward: TCGCAGCAGCACATCAACAAGAG
	Reverse: AGGTCCACGGGAAAGACACAGG
TNF-α	Forward: CGCTCTTCTGTCTACTGAACTTCGG
	Reverse: GTGGTTTGTGAGTGTGAGGGTCTG
IL-10	Forward: AAATAAGAGCAAGGCAGTGG
	Reverse: GTCCAGCAGACTCAATACACA
TGF-β1	Forward: ACCGCAACAACGCCATCTATGAG
	Reverse: GGCACTGCTTCCCGAATGTCTG

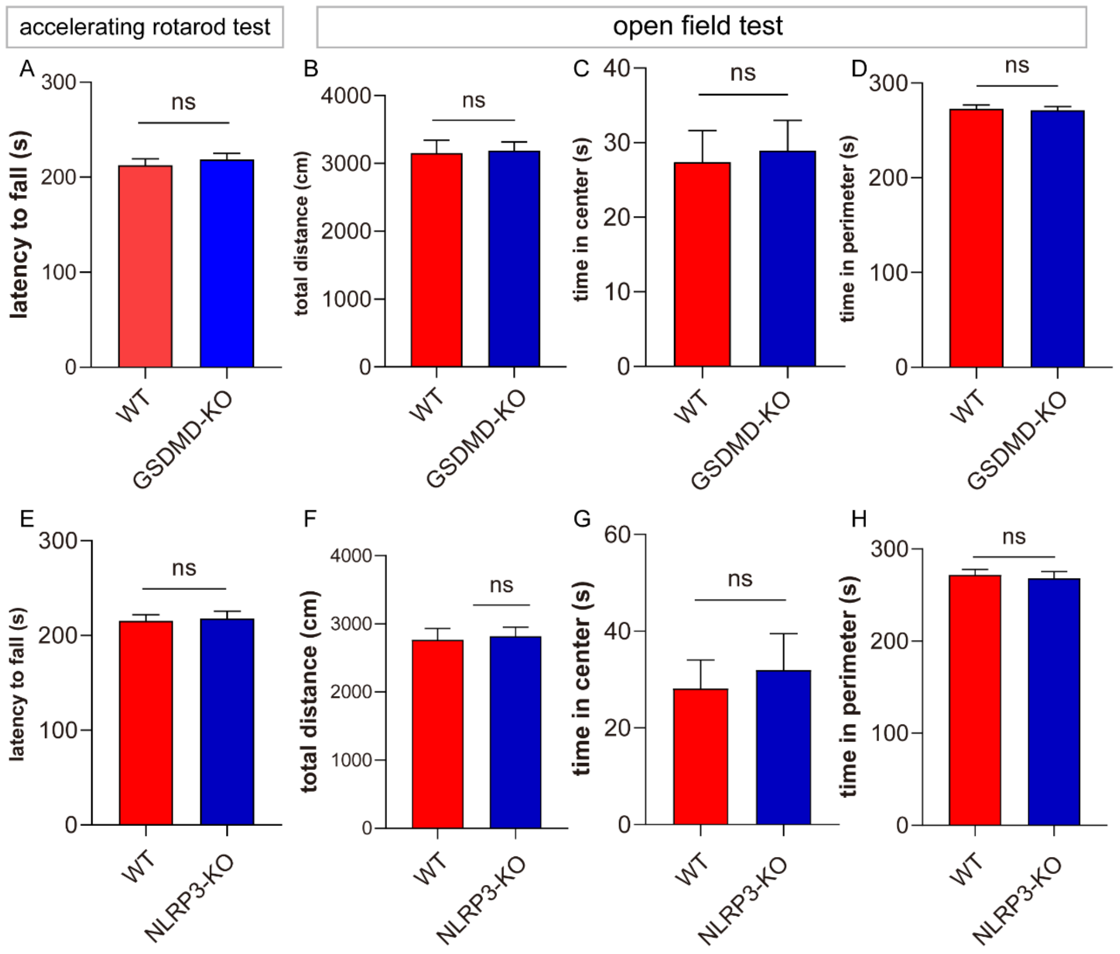

Supplementary figure S1. Results of accelerating rotarod test and open field test before TBI. (A-D) latency to fall (s), total distance (cm), time in center (s) and time in perimeter (s) in the accelerating rotarod test and open field test of the GSDMD-KO and littermate control WT mice (n=10). (E-H) latency to fall (s), total distance (cm), time in center (s) and time in perimeter (s) in the accelerating rotarod test and open field test of the NLRP3-KO and littermate control WT mice (n=10). ns p>0.05.

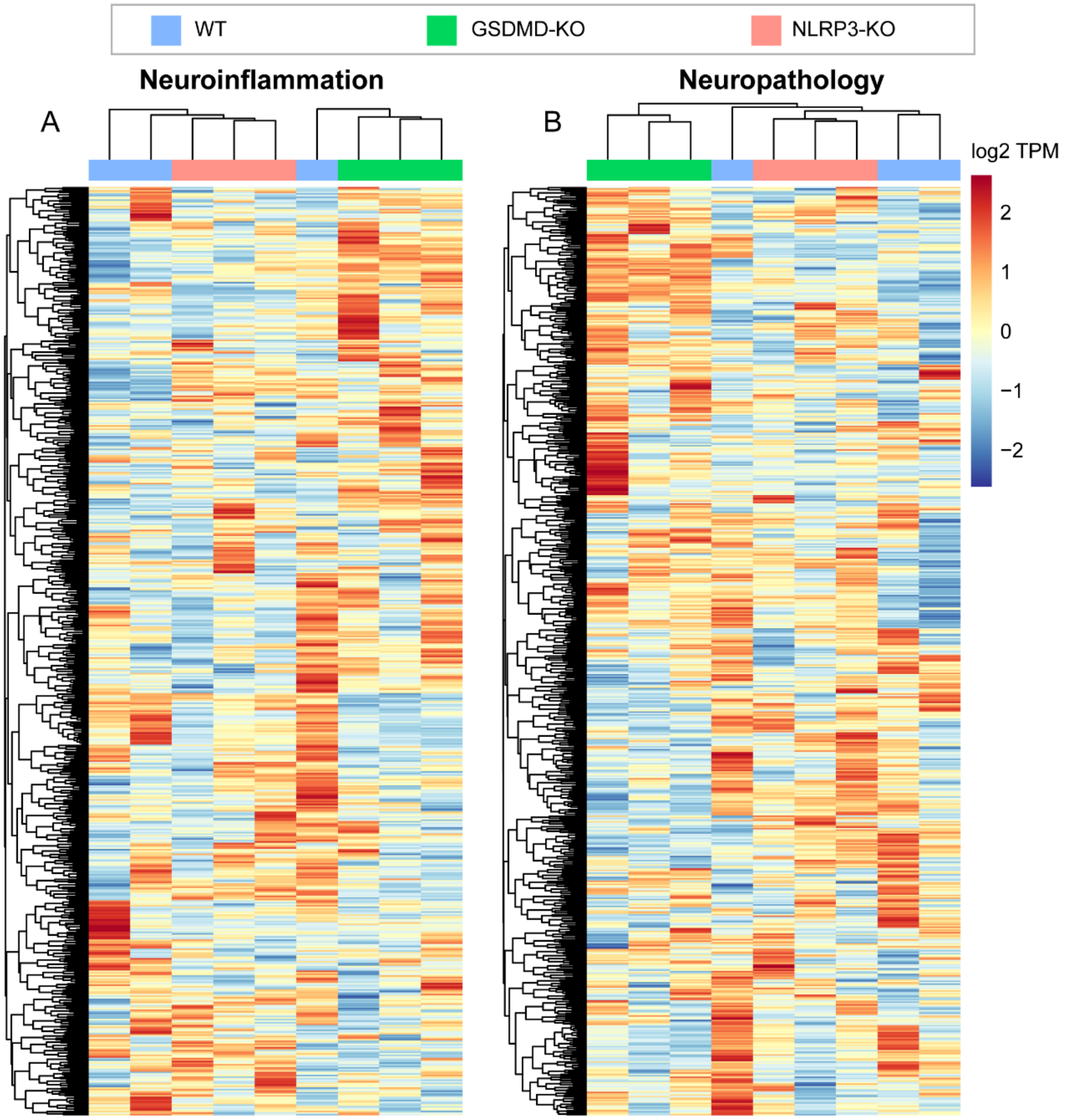

Supplementary figure S2. Heatmap analysis of neuroinflammation- and neuropathology-related gene expression of WT, GSDMD-KO and NLRP3-KO mice with sham treatment. (A-B) Expression patterns of neuroinflammation and neuropathology-related genes.
